# The industrialization of ablation: a highly standardized and reproducible workflow for radiofrequency ablation of atrial fibrillation

**DOI:** 10.1007/s10840-019-00622-y

**Published:** 2019-10-17

**Authors:** Tom De Potter, Tina D. Hunter, Lee Ming Boo, Sofia Chatzikyriakou, Teresa Strisciuglio, Etel Silva, Peter Geelen

**Affiliations:** 1 Cardiovascular Center, OLV Hospital, Moorselbaan 169, 9300 Aalst, Belgium; 2 CTI Clinical Trial and Consulting Services, Covington, KY USA; 3 Biosense Webster, Inc, Irvine, CA USA; 4 Cardiology Department, CUB-Hôpital Erasme, Université Libre de Bruxelles, Brussels, Belgium; 5 University of Naples Federico II, Naples, Italy

**Keywords:** Atrial fibrillation, Catheter ablation, Arrhythmias

## Abstract

**Background or Purpose:**

The purpose of this analysis was to report on efficacy of a standardized workflow for atrial fibrillation (AF) ablation using technology advances such as 3D imaging and contact force sensing in a real-world setting.

**Methods:**

Consecutive AF ablations from 2014 to 2015 at a high-volume site in Belgium were included. The workflow consisted of a pre-specified procedure sequence including 3D modeling followed by radiofrequency encircling of the pulmonary veins (25 W posterior wall, 35 W anterior wall) with a THERMOCOOL SMARTTOUCH® Catheter guided by CARTO VISITAG™ Module (2.5 mm/5 s stability, 50% > 7 g) and ablation index (targets: 550 anterior wall, 400 posterior wall). Efficiency endpoints were procedure time, fluoroscopy time, and radiation dose. The primary effectiveness endpoint was freedom from atrial arrhythmia recurrence.

**Results:**

A total of 605 paroxysmal AF (PAF) and 182 persistent AF (PsAF) patients were followed for 436 ± 199 days. Mean procedure times were short (PAF: 96.1 ± 26.2 min; PsAF: 109.2 ± 35.6 min) with most procedures (90.6% PAF; 81.3% PsAF) completed in ≤ 120 min. Minimal fluoroscopy was utilized (PAF: 6.1 ± 3.8 min, 5.9 ± 3.4 Gy*cm^2^; PsAF: 6.9 ± 4.7 min, 7.4 ± 4.9 Gy*cm^2^). Freedom from atrial arrhythmia recurrence was higher for PAF than PsAF patients (OR: 2.0, 95% CI: 1.4–2.9, *p* = 0.0003), but adjusted mean rates were high in both groups (81.0% vs. 67.9%). Rates were adjusted for prior ablation and age (at 65 years).

**Conclusion:**

AF ablation using a standardized workflow resulted in low procedure times and variability, with minimal fluoroscopy exposure. Long-term freedom from atrial arrhythmia recurrence was high in both PAF and PsAF populations.

## Introduction

Real-time contact force (CF)-sensing catheters have been shown to improve ablation outcomes when compared to non-CF technologies in atrial fibrillation (AF) ablation [1]. Ablation Index (AI), which integrates CF, power, and ablation time in a logarithmic formula, was shown to be an independent predictor of pulmonary vein (PV) reconnection [2]. Further, a recent study in 100 patients showed that a standardized AI-guided workflow improved 1-year outcomes compared to non-standardized CF ablation [3]. Use of these combined technologies allows for standardization of PV ablation workflow, leading to increased predictability.

The objective of this study was to assess whether the use of a highly standardized workflow, combining CF-sensing technology with AI and visualization of lesion durability, positively impacts the procedural efficiency and effectiveness of radiofrequency (RF) ablation in large real-world paroxysmal AF (PAF) and persistent AF (PsAF) populations. This study reports on improvements in the levels and variability of procedural efficiency measures—procedural duration and fluoroscopy use in particular, as well as freedom from atrial arrhythmia recurrence rates through the 12-month visit.

## Methods

This retrospective cohort study analyzed outcomes of consecutive AF ablations from a high-volume cardiovascular center in Belgium between January 2014 and December 2015. Subjects underwent RF ablation using the THERMOCOOL SMARTTOUCH® Catheter (ST; Biosense Webster, Inc.) guided by CARTO VISITAG™ Module (Visitag; Biosense Webster, Inc.) with AI. Baseline patient characteristics, procedural efficiency, and effectiveness outcomes were collected for all ablations during the study period and analyzed according to AF type (PAF vs. PsAF).

All patients gave written consent for the collection and analysis of their data. The Medical Ethics Committee of the OLV Hospital approved the use of the registry data for this study on July 13, 2016. All data used to perform the statistical analyses were de-identified and accessed in compliance with the Health Insurance Portability and Accountability Act.

### Population

Study subjects were consecutive adult patients who presented for an AF catheter ablation using an RF approach at the study site during the 2-year period. Subjects were evaluated as candidates for the procedure according to standard clinical practices with no additional criteria for study inclusion or exclusion.

### Workflow for ablations

All procedures used a 3D anatomic model generated from real-time 3D rotational angiography (3DRA) integrated automatically in the mapping system, with no additional catheter-based geometry reconstruction, using a workflow described previously and summarized below [4].

#### 3D image acquisition and integration

**Fig. 1:**
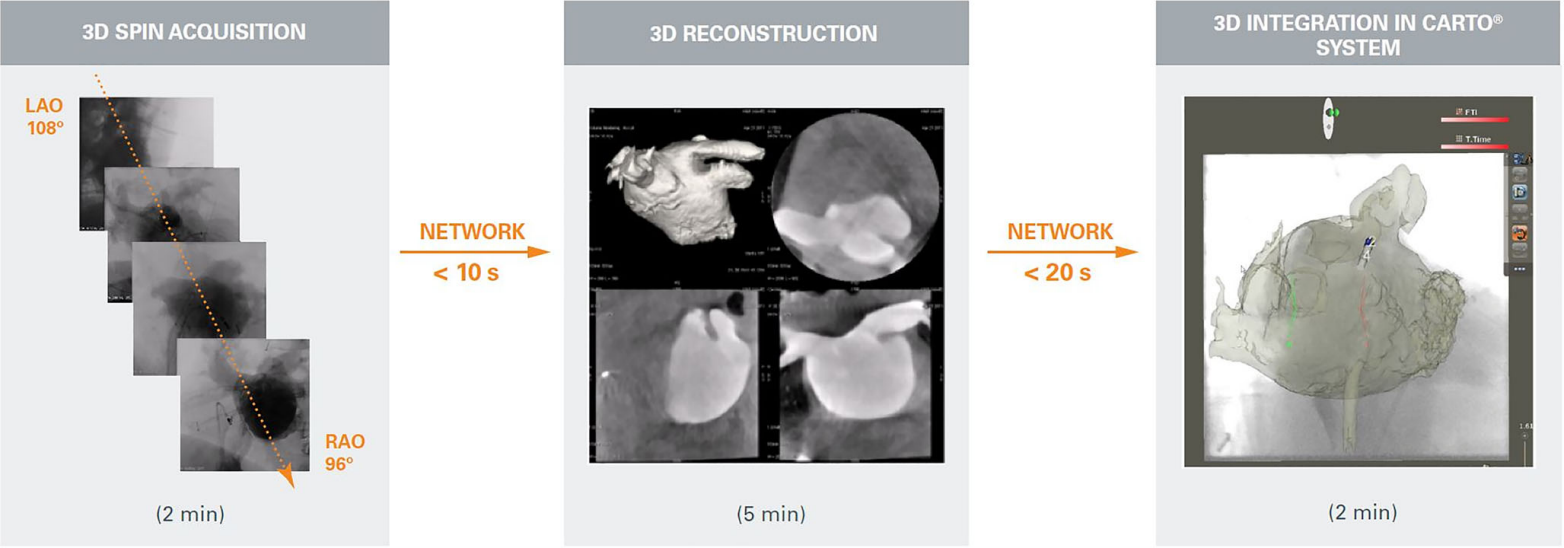
Ablation workflow schematic. *Segmentation could also be done with CARTO® “image integration” tool. In that case, the output of the 3D reconstruction can be directly retrieved from CARTO®

A 6F pigtail catheter was positioned in the center of the LA after transseptal puncture, with the pigtail positioned in the isocenter of the fluoroscopy system (GE Innova 2100) using AP and lateral projections. Prior to acquisition, the imaging system was registered to the CARTO system using the Univu module. During fast ventricular pacing (220–240 ms), 100 cc of contrast medium (Ultravist, Schering, Germany) was injected in the LA over 5 s. The C-arm was rotated around the patient during 4.5 s in an automatic sequence, acquiring near-simultaneous 2D frames during apnea. At the end of the 3DRA acquisition, the dataset was automatically transported to an imaging workstation and a 3D model was constructed using a cone beam reconstruction algorithm (Advance Workstation 3, GE). This 3D model was then transferred over the network to the CARTO workstation for integration with the electroanatomical coordinate system. Because acquisition of the 3DRA was performed with the Univu image integration functionality active, the individual background frames of the 3DRA were visible in the CARTO system in the appropriate spatial positions, allowing them to be used to visually align the 3D model with those frames. Thus, an electroanatomical shell was not required for registration, as is the case in conventional CARTOMERGE workflows. A schematic of this workflow is provided in Fig. 1.

#### Standardized ablation workflow

Pulmonary veins were isolated by sequential RF encircling with the ST catheter using this 3D model. Ipsilateral veins were ablated in pairs using a wide area circumferential ablation approach (WACA). Power settings were 25 W at the posterior wall and 35 W at the anterior wall, using recommended labeling-indicated flow rates. Standardized Visitag settings (maximum location stability range: 2.5 mm, minimum time: 5 s, and minimum force over time: 50% > 7 g) and AI settings (targeting 550 at the anterior wall and 400 at the posterior wall) were used with a maximum interlesion distance target of 8 mm.

In all *de novo* ablation patients, ablation was restricted to pulmonary vein isolation (PVI), regardless of AF type, except for patients with documented typical right atrial flutter for whom an additional cavotricuspid isthmus ablation was performed. For re-ablations, repeat PVI was performed by targeted ablation on the previously created WACA line, guided by activation patterns, and additional linear and focal ablations were performed as deemed necessary by the operator. In the case of linear lesions, bidirectional block across the line(s) was verified after ablation.

### Data collection and follow-up

Baseline patient characteristics, including pre-procedure test results, treatment history, comorbid conditions, and stroke risk scores, were recorded pre-ablation. Efficiency measures of procedure time, fluoroscopy time, and radiation dose were captured. Patients were monitored for procedure-related complications including tamponade and/or pericardial effusion, thromboembolic events, and bleeding events, but structured information was only collected prospectively and systematically beginning in 2015. Prior to 2015, only complications that led to clinical events such as death, stroke, or tamponade were collected. Beginning in 2015, active collection of subclinical events such as a hematoma was added, even when they did not lead to a new medical contact.

Follow-up visits typically occurred at 3, 6, and 12 months post-ablation, at which time patients were monitored for atrial arrhythmia recurrence via questionnaire and ECG recording, with a 24-h Holter monitor at the end of follow-up. Atrial arrhythmia recurrence included AF, atrial tachycardia, or atrial flutter. Arrhythmia events occurring in the first month after ablation were blanked from the analysis and no repeat ablations were performed within the first 3 months. Additional unscheduled Holter monitoring was performed in cases of undocumented / unexplained symptoms. Antiarrhythmic drugs (AAD), oral anticoagulation status, and AF symptoms were also recorded.

### Statistical analysis

Baseline patient characteristics, procedural efficiency measures, complications, and 12-month effectiveness outcomes were summarized by AF type. Multivariable logistic regression was used to model freedom from atrial arrhythmia recurrence through the latest follow-up visit. The explanatory variables of primary interest were baseline AF type (PAF or PsAF) and *de novo* ablation vs. re-ablation. Covariates that were tested for statistical significance included length of follow-up and baseline patient characteristics. Only variables that were statistically significant at a level of *α* = 0.10 were retained in the final model.

All statistical analyses in this study were performed using SAS software, Version 9.2 or higher (SAS Institute, Inc., Cary, NC, USA).

## Results

A total of 787 patients underwent catheter ablation for AF (605 PAF, 182 PsAF), and approximately half of these procedures were re-ablations (PAF: 46.8%, PsAF: 47.3%). There was a higher percentage of males in the PsAF group than in the PAF group (76.4% vs. 65.6%), and the PsAF patients were slightly older, at 65.4 ± 9.8 years vs. 62.7 ± 11.1 years (Table 1). Hypertension was the most prevalent comorbidity among both groups (PAF: 46.6%, PsAF: 52.8%) followed by mitral insufficiency (PAF: 31.1%, PsAF: 42.9%), which was primarily grade 1. Baseline patient characteristics are summarized in Table 1.

**Table 1:** Baseline patient characteristics

	PAF	PsAF
	(*N* = 605)	(*N* = 182)
Age, years	62.7 ± 11.1	65.4 ± 9.8
Less than 65	309 (51.1)	76 (41.8)
65–74	219 (36.2)	74 (40.7)
75 and older	77 (12.7)	32 (17.6)
Male	397 (65.6)	139 (76.4)
Body mass index kg/m^2^	27.1 ± 4.2	28.3 ± 4.5
Current smoker	46 (7.6)	27 (14.8)
Patient medical history		
Congestive heart failure	38 (6.3)	35 (19.2)
Hypertension	282 (46.6)	96 (52.8)
Diabetes	68 (11.2)	27 (14.8)
Cerebrovascular accident	43 (7.1)	10 (5.5)
End stage renal disease	3 (0.5)	2 (1.1)
CHA_2_DS_2_-VASc Score		
0–1	283 (46.8)	79 (43.4)
≥ 2	306 (50.6)	99 (54.4)
Missing	16 (2.6)	4 (2.2)
Baseline medications		
Antiarrhythmic drugs	499 (82.5)	151 (83.0)
Anticoagulation	272 (45.0)	96 (52.8)
Ejection fraction (%)	58.4 ± 7.3	53.9 ± 9.6
Collected for 2015 Procedures Only	(*N* = 327)	(*N* = 101)
Mitral insufficiency		
Grade 1	158 (26.1)	56 (30.8)
Grade 2	30 (5.0)	19 (10.4)
Grade 3	0 (0.0)	2 (1.1)
Grade 4	0 (0.0)	1 (0.6)
Left atrial size (ml)	158.9 ± 31.7	194.4 ± 42.6

Results displayed as *n* (%) or mean ± SD

*PAF:* paroxysmal atrial fibrillation, *PsAF:* persistent atrial fibrillation

### Procedural detail

Procedural efficiency measures are shown in Table 2. Mean procedure times, which included 20 min of waiting time post-PVI, were short (PAF: 96.1 ± 26.2 min; PsAF: 109.2 ± 35.6 min). The majority of procedures were performed in 120 min or less (PAF: 90.6%; PsAF: 81.3%). Mean fluoroscopy times were minimal in both the PAF (6.1 ± 3.8 min) and PsAF groups (6.9 ± 4.7 min), with corresponding radiation doses of 5.9 ± 3.4 Gy*cm^2^ and 7.4 ± 4.9 Gy*cm^2^, respectively. Acute PVI was achieved in all patients.

**Table 2:** Procedural detail

	PAF (*N* = 605)	PsAF (*N* = 182)
Ablations performed		
PVI only	539 (89.1)	155 (85.2)
PVI plus additional ablation lines	63 (10.5)	27 (14.8)
Missing	3 (0.5)	0 (0.0)
Acute pulmonary vein isolation	605 (100.0)	182 (100.0)
Total procedure time (minutes)		
Mean ± SD	96.1 ± 26.2	109.2 ± 35.6
Interquartile range (Q1, Q3)	(80, 110)	(90, 120)
Total fluoroscopy time (minutes)		
Mean ± SD	6.1 ± 3.8	6.9 ± 4.7
Interquartile range (Q1, Q3)	(3.6, 7.6)	(3.7, 8.5)
Radiation dose (Gy*cm^2^)		
Mean ± SD	5.9 ± 3.4	7.4 ± 4.9
Interquartile range (Q1, Q3)	(4.0, 6.5)	(4.4, 8.4)
Complications (captured in 2014 and 2015)		
Stroke	0 (0.0)	0 (0.0)
Cardiac tamponade	0 (0.0)	0 (0.0)
Death	0 (0.0)	0 (0.0)
Complications (captured in 2015 only)	(*N* = 333)	(*N* = 102)
Pericardial effusion	7 (2.1)	0 (0.0)
Bleeding complication	1 (0.3)	0 (0.0)

Results displayed as *n* (%) unless otherwise noted

*PAF* paroxysmal atrial fibrillation, *PsAF* persistent atrial fibrillation, *PVI* pulmonary vein isolation

Though the collection of detailed components of the total procedure time was not performed as a part of this study for all patients, a separate study collected this detail on a subset of 20 patients from the PAF cohort. A summary of the times for each step through the conclusion of the initial PV isolation is summarized in Table 3 as a point of reference for the reader.

**Table 3:** Typical times for components of ablation procedure (subset of *N* = 20 PAF ablations)

Component of procedure	Time (minutes)
Groin puncture to 1st transseptal puncture	4.5 ± 0.9
1st transseptal puncture to pigtail insertion	1.7 ± 0.7
Pigtail insertion to end of 3DRA acquisition	3.3 ± 0.7
End of 3DRA acquisition to 2nd transseptal puncture	3.2 ± 1.2
2nd transseptal puncture to 1st RF application (LPV)	9.9 ± 1.2
1st RF application (LPV) to end of LPV ablation	14.7 ± 1.6
End of LPV ablation to end of RPV ablation	15.0 ± 2.3

*3DRA:* 3D rotational angiography, *LPV:* left pulmonary vein, *RPV:* right pulmonary vein

Serious procedure-related complications were infrequent, occurring in only 1.8% (8/435) of the patients with ablations in 2015, after the structured reporting of these events began (Table 2). These consisted of seven pericardial effusion (four requiring pericardiocentesis) and one bleeding complication. There were no stroke or cardiac tamponade events, and no deaths.

### Effectiveness

**Fig. 2:**
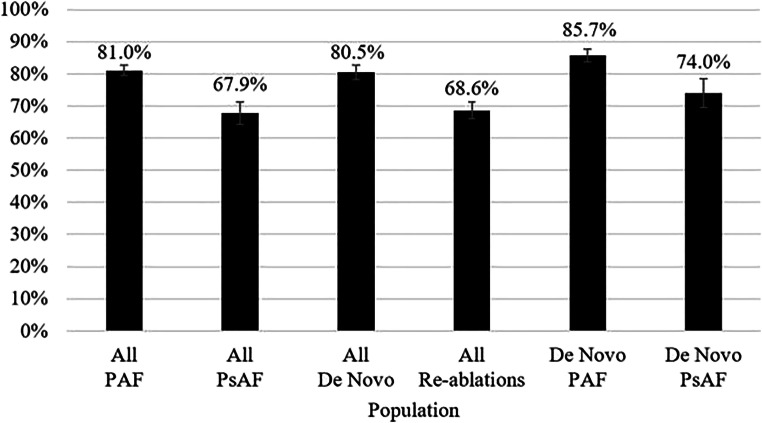
Adjusted mean rates of freedom from atrial arrhythmia recurrence. Error bars represent the standard error of the adjusted mean. Rates were calculated at 65 years of age. PAF: paroxysmal atrial fibrillation, PsAF: persistent atrial fibrillation

Rates of freedom from atrial arrhythmia recurrence through the latest follow-up visit were significantly higher for PAF patients than for PsAF patients (OR: 2.0, 95% CI: 1.4–2.9, *p* = 0.0003, Table 4). Adjusted rate estimates were 81.0 ± 1.6% for PAF vs. 67.9 ± 3.5% for PsAF (Fig. 2). Patients having *de novo* ablations had higher rates of freedom from recurrence versus those with prior ablations (OR: 1.9, 95% CI: 1.3–2.7 *p* = 0.0004, 80.5 ± 2.2% vs. 68.6 ± 2.7%). The only additional statistically significant predictor of freedom from recurrence was lower age (*p* = 0.0410), which was standardized to 65 years for calculating the adjusted mean recurrence rates.

**Table 4:** Multivariable logistic regression models of freedom from atrial arrhythmia recurrence

Variable	*P* value	Odds ratio (95% Wald confidence interval)
Full population:		
Paroxysmal AF	0.0003	2.02 (1.38, 2.95)
*De novo* AF ablation	0.0004	1.88 (1.33, 2.67)
Age (per year)	0.0410	0.98 (0.97, 1.00)
*De novo* ablations only:		
Paroxysmal AF	0.0080	2.11 (1.22, 3.67)

*AF:* atrial fibrillation

In the subset of 418 patients (322 PAF, 96 PsAF) with *de novo* ablations, first procedure freedom from recurrence rate estimates were higher in both cohorts than those seen in the respective full population cohort (PAF:85.7 ± 2.0%, PsAF: 74.0 ± 4.5%, Fig. 2).

Re-ablation rates were 9.6% and 9.9% over mean follow-up times of 438 ± 201 days and 430 ± 190 days in the PAF and PsAF cohorts, respectively. AAD utilization was ongoing in 34.7% and 40.1% of the PAF and PsAF cohorts as of their last follow-up visit.

## Discussion

The current analysis suggests that using a standardized AI-guided workflow and integrated 3D angiography-derived models enables predictable procedural efficiency. Procedure and fluoroscopy times under the standardized workflow were markedly shorter and less variable when compared to prior studies of CF-sensing technology [1, 5]. For example, comparing the PAF cohort in our study to the published SMART-AF cohort, a > 2-fold average procedure time reduction was observed (96 min vs. 222 min) [5]. Perhaps even more important from a standardization perspective is the observed reduction in the variability of procedure times in a real-world setting of consecutive cases (standard deviation of procedure time: 26 min vs. 84 min in SMART-AF) [5].

Serious procedure-related complications were infrequent and within previously reported ranges [5], indicating that efficiency and effectiveness gains did not compromise patient safety. Freedom from atrial arrhythmia recurrence among PAF patients was higher than typical of previously published studies on CF-sensing technology [5]. Although procedural efficiency and effectiveness were greater in the PAF cohort compared to the PsAF cohort, the PsAF cohort also demonstrated good efficiency and long-term effectiveness, consistent with previous reporting of CF ablation compared to pre-CF ablation technology [6].

Despite improvements in clinical outcomes associated with the use of CF-sensing catheters and integrated electroanatomic mapping technologies [1, 7], procedure and fluoroscopy times can still be lengthy, and radiation exposure remains a concern for both patients and healthcare professionals [8]. Prior to the AI-facilitated standardized workflow, procedure times at the study site varied widely from case to case and were generally between 90 and 120 min on the low end and 4–5 h on the high end. The improved efficiency, reflected in the predictably shorter procedure and fluoroscopy times seen in this analysis of AI-guided ablation, underscores the importance of recent advancements in CF technology that enable standardization of PVI workflow. The shorter and less variable procedure times could potentially translate to improved procedure scheduling and long-term cost savings [9]. Another strategy for shortening procedure time that has garnered significant attention over the past year is ablation with high power and short duration (HPSD), defined as 40-50 W with current catheters or up to 90 W with novel catheters that are currently in development [10–15]. If proven, HPSD ablation could further reduce the procedure time via a reduction in the time required for RF application.

In the presented workflow, real-time 3D modeling was derived from rotational angiography and the automatic integration of this modeling in the 3D mapping system allowed for drastic reduction in fluoroscopy use, all but eliminating the need for fluoroscopy during the ablation phase. When using a 3D mapping system, there was previously no way to position an external 3D model (e.g., from CT or MRI) within the coordinate system of the mapping model. This limitation has been overcome by creating a catheter-based 3D model, then registering the external model with landmarks and surface fitting. Importantly, our workflow does not require any additional mapping because the coordinates of the 3DRA model are linked to the CARTO system through the Univu module. Therefore, the time and fluoroscopy associated with 3D geometry creation, which varies depending on anatomy and operator experience, is eliminated. All steps required for the 3DRA processing, other than the acquisition (1 min total), are performed by a technician in parallel with ongoing steps of the procedure such that they do not impact overall procedure time.

If operators work with only the 3D model generated by the mapping system, the advantages of the workflow described above will obviously not apply, but there will be a different set of limitations. On top of the time and experience needed for acquisition of the 3D model by catheter manipulation, there is almost always a need for manual adjustment of these models (i.e., erasing certain regions, smoothening, adjusting mapping resolution) in order to obtain a high quality 3D representation. We believe that the 3DRA workflow presented here offers the best of both worlds by allowing a highly detailed 3D model to be acquired and integrated in the mapping system without manipulation, adjustment or fine-tuning.

The presented numbers reflect the fluoroscopy needed for procedural set-up (catheter placement, transseptal puncture) and 3D acquisition. Fluoroscopy exposure was primarily driven by the 3D angiography acquisition, during which the operator was outside of the room and not exposed to radiation. Therefore, typical operator exposure doses were well below 1 microSievert during the PVI procedure per real-time electronic dosimeter recordings (data not presented here). Alternatively, similarly low exposure rates could be achieved with the use of intracardiac echocardiography. However, this modality requires time and operator experience and is associated with prohibitively high costs in most healthcare reimbursement models outside the US.

Our results demonstrate that both PAF and PsAF patients had lower recurrence rates than those commonly seen among comparable groups receiving RF ablation [5, 16, 17]. A recent study from a single center using a similar standardized AI workflow showed similar results with an overall single procedure success rate of > 90% in PAF patients [3, 18]. In addition to an AI target value, the aforementioned study also utilized an interlesion distance of ≤ 6 mm to ensure lesion contiguity around the PV. Our study used a target interlesion distance of ≤ 8 mm based on an earlier retrospective analysis of our site data.

Given that PVI is the cornerstone for all AF ablation, it is possible that AI facilitates stable catheter-tissue contact for consistent and more durable lesion creation around the PVs, thereby improving subsequent long-term outcomes. The associations of catheter stability and lesion contiguity with improved long-term success have been previously demonstrated [19, 20]. It will be important to continue observing real-world clinical outcomes as more data become available from standardized AI protocols.

### Limitations

The primary limitations of this study are consequences of the non-randomized retrospective design from a single site. In particular, the results may lack generalizability due to site-specific factors such as procedural workflow, level of operator experience and skill, or unique characteristics of the patient population. Procedure-related complications were only systematically and prospectively collected since 2015, such that subclinical complications occurring prior to 2015 may have been missed. Furthermore, there is potential that unmeasured variables related to either the patients or procedures could confound the presented results. Finally, as we did not formally compare our approach to other workflows, we cannot offer proof that this particular workflow has superior characteristics in terms of procedural time or variability.

## Conclusion

A highly standardized workflow for AF ablation greatly reduced procedural variability by using 3D rotational angiography and image integration for the mapping phase, and Visitag-AI guided ablation enabled by the ST CF-sensing catheter for the ablation phase. This workflow led to predictably low procedure and fluoroscopy times, with good clinical outcomes as seen in the high rates of freedom from atrial arrhythmia recurrence in both PAF and PsAF populations.
